# Quality and content evaluation of anal fistula information on TikTok and Bilibili: A cross-sectional study

**DOI:** 10.1097/MD.0000000000050130

**Published:** 2026-08-14

**Authors:** Xizhe Du, Shaopeng Huang, Qinghua Ye, Shuting Pan, Xia Xiao, Jiayi Xia, Yingru Zhuang

**Affiliations:** a Department of Anorectal Surgery, Shantou Hospital of Traditional Chinese Medicine, Guangzhou University of Chinese Medicine, Shantou, China.

**Keywords:** anal fistula, Bilibili, health communication, reliability, short videos, TikTok, video quality

## Abstract

Short-video platforms are widely used for medical information, but the quality of anal fistula content remains uncertain. We evaluated anal fistula-related videos on TikTok and Bilibili and compared platforms and uploader categories. The top 200 videos under each platform’s default comprehensive ranking were screened. Eligible videos were assessed using the Global Quality Score (GQS), modified DISCERN (mDISCERN), Journal of the American Medical Association benchmark criteria (JAMA), and 7-indicator completeness score (7-ICS). Platform and uploader differences were evaluated using nonparametric tests. Spearman correlations examined relationships between engagement and quality, and multivariable ordinal logistic regression identified factors associated with higher scores. Of 400 screened videos, 333 were included (TikTok, 167; Bilibili, 166). Median GQS, mDISCERN, JAMA, and 7-ICS scores were each 2.00. Treatment (71.4%) and clinical manifestations (63.9%) were the most frequently covered domains, whereas epidemiology (7.2%) and prevention (12.6%) were least covered. TikTok videos were shorter and received greater engagement than Bilibili videos (all *P* < .001). Bilibili had higher unadjusted 7-ICS scores and JAMA score distributions, while GQS and mDISCERN did not differ. After adjustment, TikTok was associated with higher GQS (OR, 2.26; 95% CI: 1.27–4.02) and lower 7-ICS (OR, 0.26; 95% CI: 0.13–0.50), but not with JAMA or mDISCERN. Specialist-physician uploader status and longer video duration were associated with higher scores for all 4 outcomes. Engagement metrics were strongly intercorrelated but were generally unrelated to quality measures. Anal fistula-related videos on both platforms were generally incomplete and of suboptimal quality. Platform differences were mixed rather than uniformly favoring TikTok or Bilibili. Specialist-physician authorship and longer duration were consistently associated with better scores. These cross-sectional findings represent associations, not causal effects.

## 1. Introduction

Anal fistula, also known as perianal fistula, is an abnormal epithelium-lined tract connecting the anal canal with the perianal skin.^[1,2]^ It is a common anorectal disorder, with a higher prevalence in males than females and peak incidence between 20 and 40 years of age.^[3,4]^ Anorectal abscess is the most frequent underlying cause. Among patients with anorectal abscess, 30% to 70% have a concurrent anal fistula, and approximately one-third of those without an initial fistula may develop 1 within months to years after abscess drainage.^[5,6]^ Patients may experience pain, difficulty sitting, purulent or bloody discharge, and, in severe cases, systemic sepsis.^[7]^ Although usually benign, anal fistula can cause substantial physical discomfort, psychological distress, and impaired quality of life.^[6,8]^ Studies using the anal fistula Quality of Life Scale have shown that both physical symptoms and psychological burden significantly affect patients’ well-being.^[9]^ Therefore, early recognition, timely intervention, and improved public awareness are important for reducing disease burden.

Short-video platforms have rapidly become important channels for health information dissemination. TikTok, known as Douyin in China, and Bilibili are widely used social media platforms through which the public increasingly obtains medical information.^[10,11]^ Accurate and evidence-based online health information may reduce anxiety, improve treatment adherence, and support quality of life, whereas misleading content may increase health risks and contribute to inappropriate medical decisions.^[12-14]^ Therefore, evaluating the quality of medical videos on these platforms is important. Although previous studies have assessed short-video information on digestive diseases, including colorectal cancer, irritable bowel syndrome, and inflammatory bowel disease,^[15]^ evidence regarding anal fistula-related videos remains limited. Given the clinical burden of anal fistula^[3,4]^ and the increasing participation of specialist physicians in online health communication,^[16]^ this study conducted a cross-sectional analysis of anal fistula-related videos on TikTok and Bilibili. We aimed to compare video characteristics, engagement, content coverage, and quality across platforms and uploader groups, and to identify factors associated with higher-quality medical information.

## 2. Materials and methods

### 2.1. Ethical considerations

The Ethics Committee of Shantou Hospital of Traditional Chinese Medicine waived the requirement for ethical approval and informed consent because this study used only publicly available online videos and did not involve human participants, clinical records, intervention, or identifiable private patient information.

### 2.2. Video collection

This cross-sectional study systematically gathered short-video data related to the Chinese search term “肛瘘” (“anal fistula”) on Bilibili (https://www.bilibili.com) and TikTok/Douyin (https://www.douyin.com) from August 20 to 23, 2025. On August 19, 2025, the researchers created a new independent account for each platform. These accounts had no prior user activity records (e.g., search history, likes, or follows) or professional certifications (e.g., medical or enterprise certification). Personalized recommendation settings were disabled where available. Searches were conducted between 08:00 and 12:00 UTC. To further minimize personalization bias, browser caches and cookies were cleared, and the virtual location was set to Shantou, China.

The search results generated video lists based on the platforms’ default comprehensive search strategy. We chose the default comprehensive ranking for 2 specific reasons relevant to this study. First, default comprehensive search is considered the primary method by which users retrieve videos.^[17,18]^ This ranking reflects the actual conditions encountered by average users, as most users do not modify the default sorting settings. Therefore, sampling from this ranking captures the information environment most directly and widely accessed by the public, aligning with our research objective to evaluate the quality of content that average users are likely to find.^[19]^ Second, the “Comprehensive” ranking utilizes an integrated algorithm that considers factors such as relevance, interaction, timeliness, and author authority, thereby providing a broad overview of popular content on the topic as curated by the platform.^[20]^ No additional filters were applied during the search process to ensure the objectivity of the initial sample selection.

Consistent with several previous studies that included the top 200 videos under the default sorting of a single platform,^[15,18,21]^ the sample size of 200 videos per platform in this study was determined based on preliminary observations and analytical feasibility. Preliminary search and browsing behavior simulated by our research team indicated that the relevance and completeness of video content related to the search term “anal fistula” significantly declined after the first few pages of results (corresponding approximately to the top 150–200 videos), while content duplication and low relevance became more prevalent. Therefore, this study adopted a sample size of 200 videos per platform, aiming to capture the core, prominent, and easily accessible content encountered by typical users during the search process while maintaining a manageable sample size for in-depth quality assessment. After an initial screening of 400 videos from both platforms, videos meeting the eligibility criteria were included in the final analysis.

**Figure 1.**
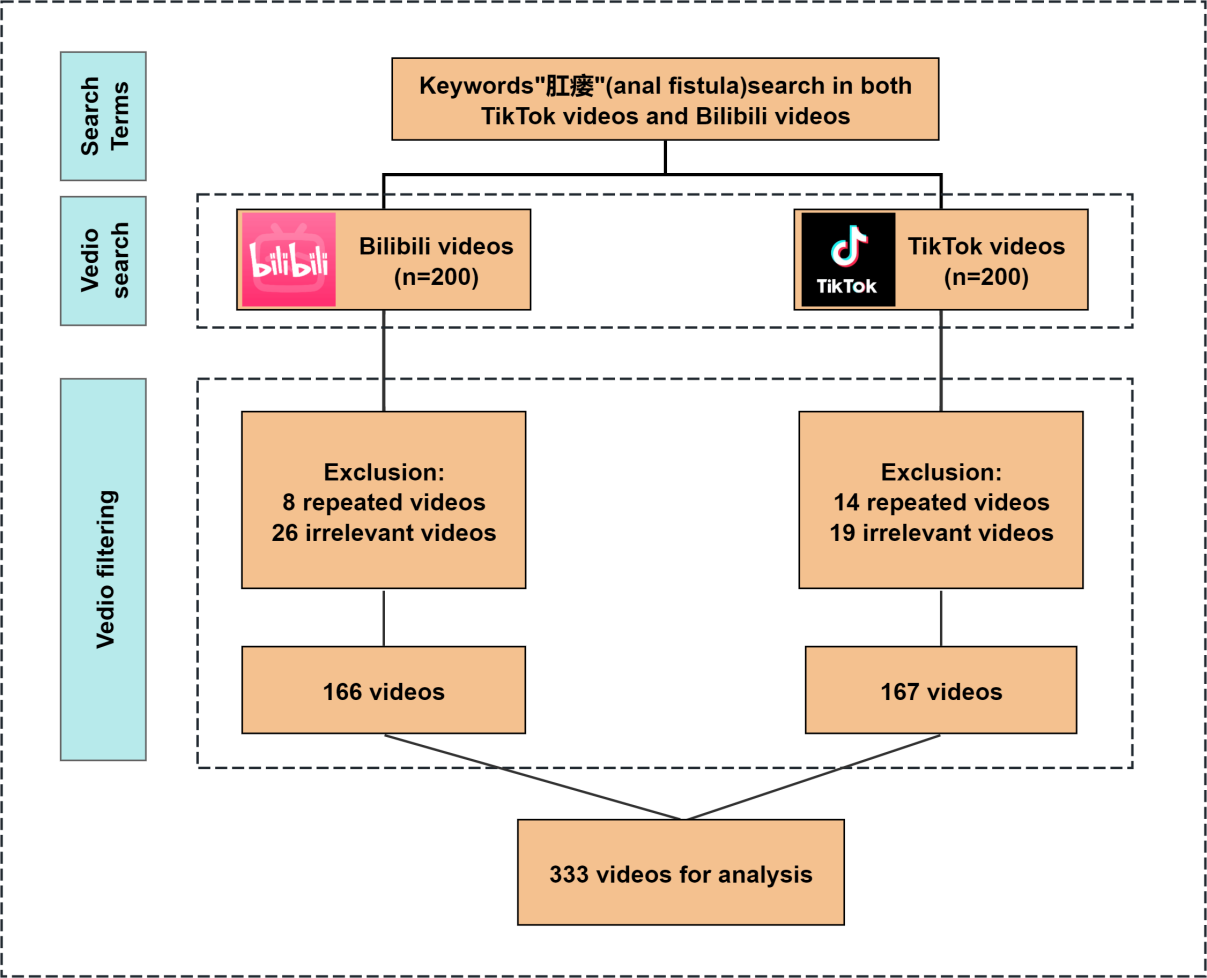
Flow diagram of video identification, screening, and inclusion.

The 400 retrieved videos then underwent eligibility screening. Duplicate videos and videos irrelevant to anal fistula were excluded. On TikTok, 14 repeated and 19 irrelevant videos were excluded, leaving 167 videos; on Bilibili, 8 repeated and 26 irrelevant videos were excluded, leaving 166 videos. The final sample therefore comprised 333 videos (Fig. 1). General information – including video title, URL, uploader name, likes, collections, shares, comments, and video duration – was recorded in an Excel spreadsheet (Microsoft Corp, Redmond).

### 2.3. Uploader characteristics

Video uploaders were categorized into 4 groups: specialist physicians, defined as physicians with formal medical qualifications and specialization in colorectal or anorectal diseases; non-specialist physicians, defined as physicians with formal medical qualifications but without specialization in anorectal diseases; institutions, including organizations primarily focused on medical education, disease awareness, or public health communication; and patients, including individuals currently affected by anal fistula or with a history of the condition. For the binary comparison, these categories were collapsed into specialist-physician uploaders and other uploaders. The latter group included non-specialist physicians, institutions, and patients. The term “other uploaders” was used instead of “nonprofessionals” because this group included non-specialist physicians and institutions with medical or health-related backgrounds, in addition to patients.

### 2.4. Quality and reliability evaluation of videos

To assess the quality and reliability of the videos, we utilized 4 rating tools: Global Quality Score (GQS),^[22]^ the modified DISCERN scale (mDISCERN),^[23]^ Journal of the American Medical Association (JAMA) benchmark criteria,^[24]^ and the 7-indicator completeness score (7-ICS). These tools are widely recognized for evaluating the quality of health information. The GQS assesses online video quality across 5 levels, ranging from 1 (poor) to 5 (excellent) (Table S1, Supplemental Digital Content 1). The mDISCERN scale, originally designed for written health information and later modified for video content, includes 5 questions with a total score ranging from 0 to 5, with higher scores indicating greater reliability (Table S2, Supplemental Digital Content 2). The JAMA benchmark criteria evaluate various aspects, such as authorship, citation of sources, timeliness, and disclosure of conflicts of interest, and were used in this study to further assess the quality and reliability of videos (Table S3, Supplemental Digital Content 3).

We used a comprehensive content integrity checklist, initially developed by Zhang et al for colorectal cancer videos,^[25]^ and further refined by Zhu et al into the Completeness Score.^[26]^ It retained core domains, such as Etiology, Symptoms, Prevention, Treatment, and Prognosis, while merging “Anatomy” into “Etiology and Risk Factors,” resulting in 5 mutually exclusive categories for clarity. Tu et al highlighted that a high-quality short video should cover all aspects of a disease, including etiology, clinical symptoms, diagnosis, treatment, rehabilitation, and prevention.^[27]^ Sarveazad et al emphasized that epidemiological data on anal fistulas are crucial for developing optimal policies for control and treatment.^[28]^ Based on the CS, we added “Epidemiology” and “Diagnosis,” leading to the 7-ICS, which includes Epidemiology, Etiology, Clinical Manifestations, Diagnosis, Treatment, Prevention, and Prognosis. Each domain was scored 0 if not mentioned and 1 if mentioned. The 7-ICS ranged from 0 to 7.

The ratings were independently completed by 2 colorectal specialists. In cases of significant disagreement, the final decision was made by the chief colorectal physician. All assessors underwent standardized training prior to scoring to ensure consistency and minimize bias.

### 2.5. Video assessment and inter-rater reliability

Video evaluations were conducted independently by 2 senior colorectal surgeons specializing in anal fistula (reviewer A: PST; reviewer B: XX). Both reviewers are affiliated with the Colorectal Surgery Department of a tertiary-level traditional Chinese medicine hospital and possess over 10 years of clinical experience in the diagnosis and management of anal fistula. To ensure consistency in interpretation, the reviewers thoroughly studied the operational definitions and detailed instructions for the mDISCERN instrument, GQS, JAMA, and 7-ICS. Calibration was performed using a set of sample videos not included in the final analysis. The 2 reviewers independently scored these videos and subsequently discussed any scoring discrepancies during consensus meetings. Ambiguous scoring criteria and content coding rules were clarified during the calibration process. After achieving satisfactory inter-rater consistency, each reviewer independently assessed all 333 videos and documented the scores for the 4 evaluation tools. In cases of scoring discrepancies, the 2 reviewers first compared their individual assessments to identify the source of disagreement. If consensus could not be reached, a third reviewer – a colorectal surgery expert with over 35 years of experience in anal fistula management (ZYR) – was consulted. This senior expert reviewed the video and both initial evaluations, facilitated a discussion, and finalized the score based on alignment with clinical guidelines and evidence-based practice. Inter-rater reliability was quantified using intraclass correlation coefficients (ICCs), which were interpreted as follows: poor (ICC < 0.50), moderate (0.50 ≤ ICC < 0.75), good (0.75 ≤ ICC < 0.90), or excellent (ICC ≥ 0.90).

All 333 videos were included in the inter-rater reliability analyses. Agreement was excellent for GQS (ICC = 0.913, 95% CI: 0.891–0.930), mDISCERN (ICC = 0.900, 95% CI: 0.873–0.922), 7-ICS (ICC = 0.907, 95% CI: 0.884–0.925), and JAMA (ICC = 0.905, 95% CI: 0.884–0.923).

### 2.6. Statistical analysis

The normality of continuous variables was evaluated using the Shapiro–Wilk test. Normally distributed data are presented as mean ± standard deviation (SD), whereas non-normally distributed data are presented as median and interquartile range (IQR). Differences between 2 groups were analyzed using the Mann–Whitney *U* test. For comparisons involving 3 or more groups, the Kruskal–Wallis test was used, followed by Dunn’s post hoc test with Holm correction. Spearman rank correlation coefficients (ρ) were calculated to evaluate associations between video length, engagement metrics (likes, collections, comments, and shares), and quality or reliability scores. Correlation strength was classified as follows: |ρ| < 0.2, negligible; 0.2 to 0.4, weak; 0.4 to 0.6, moderate; 0.6 to 0.8, strong; and > 0.8, very strong. Multivariable ordinal logistic regression models were constructed with GQS, JAMA, mDISCERN, and 7-ICS as dependent variables. The primary models included platform, binary uploader classification, video length, and engagement metrics; secondary models replaced the binary uploader variable with the 4-category uploader classification. No data were missing for the variables included in the analyses. Statistical significance was defined as a 2-sided *P* < 0.05. All analyses were performed using R (version 4.4.0; R Foundation for Statistical Computing).

## 3. Results

### 3.1. Video characteristics

After screening, 333 videos were included (TikTok, 167; Bilibili, 166). Bilibili videos were longer, whereas TikTok videos received more likes, collections, comments, and shares (all *P* < .001; Table 1). Specialist-physician videos received more likes, collections, and shares than videos from other uploaders, while video length and comments did not differ (Table 2). Across the 4 uploader categories, all engagement variables differed overall. Patient-generated videos received more comments than most other categories, and specialist-physician videos received more likes than institutional videos and more collections than patient videos. Detailed post hoc comparisons are provided in Table S4, Supplemental Digital Content 4.

**Table 1:** Video characteristics, content coverage, and quality scores on TikTok and Bilibili.

Variables	Total (n = 333)	Bilibili (n = 166)	TikTok (n = 167)	*P*
General information				
Video length, M (Q_1_, Q_3_)	60.00 (33.00, 126.00)	79.50 (47.00, 189.75)	46.00 (15.00, 88.50)	<.001
Likes, M (Q_1_, Q_3_)	272.00 (37.00, 1528.00)	59.00 (11.00, 583.75)	670.00 (218.50, 2455.50)	<.001
Collections, M (Q_1_, Q_3_)	71.00 (9.00, 355.00)	17.50 (4.00, 130.75)	215.00 (36.00, 517.50)	<.001
Comments, M (Q_1_, Q_3_)	127.00 (13.00, 456.00)	19.50 (2.00, 143.50)	391.00 (126.00, 967.00)	<.001
Shares, M (Q_1_, Q_3_)	70.00 (8.00, 693.00)	10.50 (2.00, 90.50)	324.00 (55.00, 1612.00)	<.001
Content				
Epidemiology	24 (7.2%)	9 (5.4%)	15 (8.9%)	-
Etiology	114 (34.2%)	55 (33.1%)	59 (35.3%)	-
Clinical manifestation	213 (63.9%)	120 (72.2%)	93 (55.6%)	-
Diagnosis	122 (36.6%)	60 (36.1%)	62 (37.1%)	-
Treatment	238 (71.4%)	134 (80.7%)	104 (62.2%)	-
Prevention	42 (12.6%)	21 (12.6%)	21 (12.5%)	-
Prognosis	121 (36.3%)	81 (48.7%)	40 (23.9%)	-
Quality and reliability				
7-ICS, M (Q_1_, Q_3_)	2.00 (1.00, 4.00)	3.00 (2.00, 4.00)	2.00 (1.00, 3.50)	<.001
GQS, M (Q_1_, Q_3_)	2.00 (1.00, 3.00)	2.00 (2.00, 3.00)	2.00 (1.00, 3.00)	.706
mDISCERN, M (Q_1_, Q_3_)	2.00 (1.00, 2.00)	2.00 (1.00, 2.00)	2.00 (1.00, 2.00)	.191
JAMA score, M (Q_1_, Q_3_)	2.00 (2.00, 3.00)	2.00 (2.00, 3.00)	2.00 (1.00, 3.00)	<.001

7-ICS = 7-indicator completeness score, GQS = Global Quality Score, JAMA = Journal of the American Medical Association, M = median, mDISCERN = modified DISCERN, Q1 = first quartile, Q3 = third quartile,.

**Table 2:** Video characteristics and quality scores for specialist-physician and other uploaders.

Variables	Total (n = 333)	Other uploaders (n = 160)	Specialist-physician uploaders (n = 173)	*P*
General information				
Video length, M (Q_1_, Q_3_)	60.00 (33.00, 126.00)	80.50 (15.00, 180.25)	54.00 (37.00, 89.00)	.117
Likes, M (Q_1_, Q_3_)	272.00 (37.00, 1528.00)	145.50 (29.75, 699.75)	617.00 (56.00, 1973.00)	.008
Collections, M (Q_1_, Q_3_)	71.00 (9.00, 355.00)	34.00 (8.00, 236.00)	121.00 (13.00, 477.00)	.008
Comments, M (Q_1_, Q_3_)	127.00 (13.00, 456.00)	186.50 (22.50, 537.50)	101.00 (10.00, 421.00)	.267
Shares, M (Q_1_, Q_3_)	70.00 (8.00, 693.00)	36.50 (7.00, 282.50)	148.00 (8.00, 1219.00)	.004
Quality and reliability				
7-ICS, M (Q_1_, Q_3_)	2.00 (1.00, 4.00)	2.00 (1.00, 3.00)	3.00 (2.00, 4.00)	<.001
GQS, M (Q_1_, Q_3_)	2.00 (1.00, 3.00)	2.00 (1.00, 2.00)	2.00 (2.00, 3.00)	<.001
mDISCERN, M (Q_1_, Q_3_)	2.00 (1.00, 2.00)	1.00 (1.00, 2.00)	2.00 (2.00, 3.00)	<.001
JAMA score, M (Q_1_, Q_3_)	2.00 (2.00, 3.00)	2.00 (1.00, 2.00)	2.00 (2.00, 3.00)	<.001

7-ICS = 7-indicator completeness score, GQS = Global Quality Score; JAMA = Journal of the American Medical Association; M = median, mDISCERN = modified DISCERN, Q1 = first quartile; Q3 = third quartile.

### 3.2. Uploader characteristics

**Figure 2.**
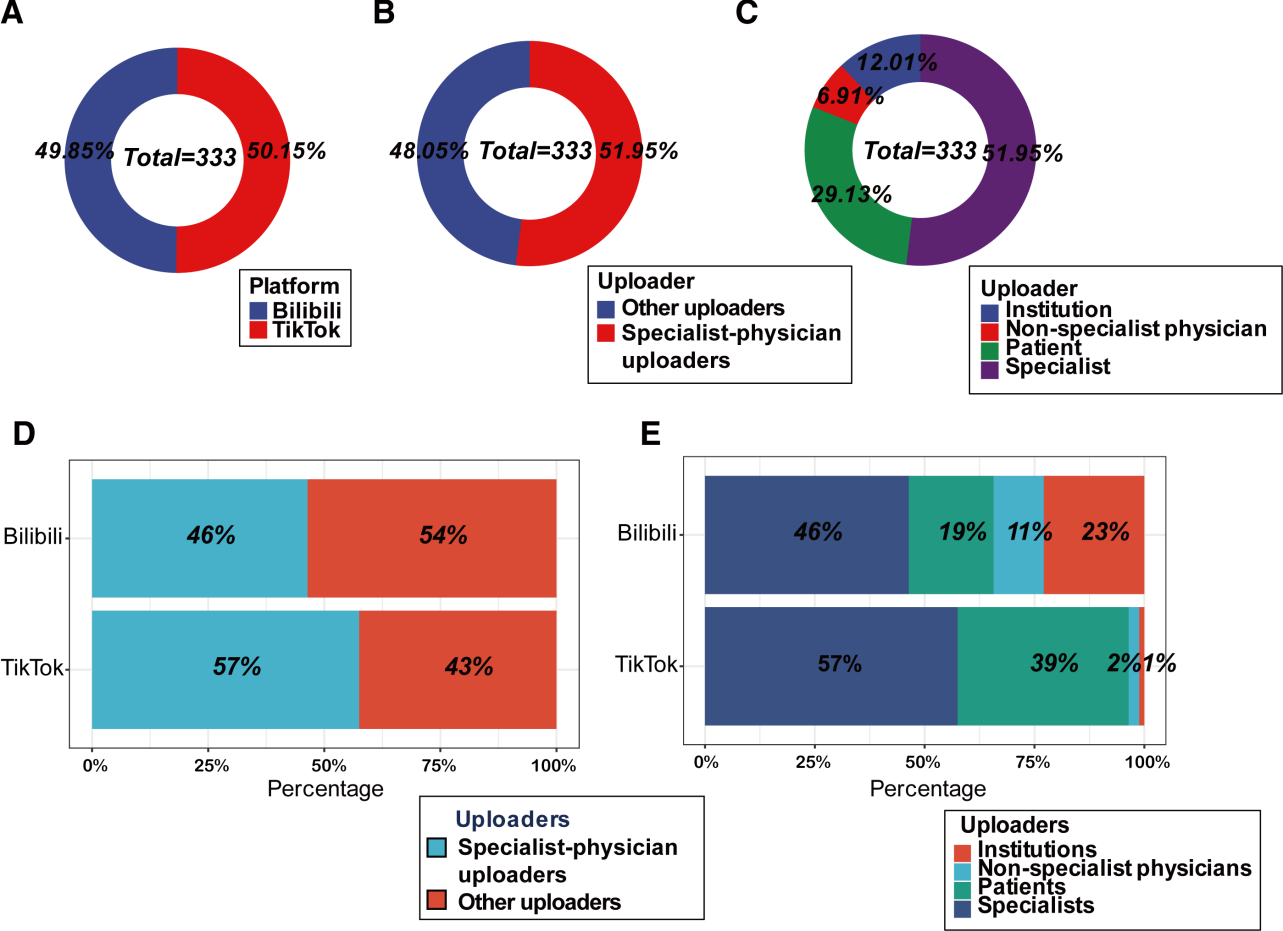
Distribution of platforms and uploader categories. (A) Overall platform distribution. (B) Overall distribution of specialist-physician and other uploaders. (C) Overall distribution of the 4 uploader categories. (D) Distribution of specialist-physician and other uploaders by platform. (E) Distribution of the 4 uploader categories by platform.

The platform distribution was nearly equal (Bilibili, 49.85%; TikTok, 50.15%; Fig. 2A). Specialist physicians contributed 173 videos (51.95%), followed by patients (29.13%), institutions (12.01%), and non-specialist physicians (6.91%; Fig. 2B, C). Uploader distributions differed between platforms: TikTok contained higher proportions of specialist-physician and patient videos, whereas Bilibili contained higher proportions of institutional and non-specialist-physician videos (Fig. 2D, E).

### 3.3. Video content

Treatment (71.4%) and clinical manifestations (63.9%) were the most frequently covered domains, whereas epidemiology (7.2%) and prevention (12.6%) were least frequently covered. Bilibili more frequently covered clinical manifestations, treatment, and prognosis than TikTok (Table 1 and Figure S1A, Supplemental Digital Content 5).

### 3.4. Video quality

Overall quality was low, with median GQS, mDISCERN, JAMA, and 7-ICS scores of 2.00 (Table 1). All videos met the JAMA currency criterion, but only 1 video provided attribution; authorship and disclosure were also inconsistently reported (Fig. S1B–D, Supplemental Digital Content 5). Unadjusted platform findings were mixed: Bilibili had higher 7-ICS scores and a higher JAMA score distribution, whereas GQS and mDISCERN did not differ between platforms (Table 1).

Specialist-physician videos had higher scores than videos from other uploaders for all 4 outcomes (all *P* < .001; Table 2). Across the 4 uploader categories, patient-generated videos scored lower than each of the other categories on all 4 measures, while specialist-physician videos also had higher JAMA scores than institutional videos. No other pairwise differences among specialist physicians, non-specialist physicians, and institutions were significant for 7-ICS, GQS, or mDISCERN (Table 3).

**Table 3:** Video quality, reliability, and content completeness across 4 uploader categories.

Uploader	Variables			
	7-ICS, M (Q_1_, Q_3_)	GQS, M (Q_1_, Q_3_)	mDISCERN, M (Q_1_, Q_3_)	JAMA score, M (Q_1_, Q_3_)
Institution(n = 40)	3.00 (2.00, 4.00)	2.00 (2.00, 3.00)	2.00 (2.00, 2.00)	2.00 (2.00, 2.00)
Non-specialist physician(n = 23)	4.00 (2.50, 4.00)	2.00 (2.00, 3.00)	2.00 (2.00, 3.00)	2.00 (2.00, 2.00)
Patient(n = 97)	1.00 (1.00, 2.00)	1.00 (1.00, 2.00)	1.00 (1.00, 1.00)	1.00 (1.00, 2.00)
Specialist(n = 173)	3.00 (2.00, 4.00)	2.00 (2.00, 3.00)	2.00 (2.00, 3.00)	2.00 (2.00, 3.00)
P*	<.001	<.001	<.001	<.001
*P*ΔI vs N	.742	1	.496	.310
*P*ΔI vs P	<.001	<.001	<.001	.005
*P*ΔI vs S	.742	.645	.177	<.001
*P*ΔN vs P	<.001	<.001	<.001	<.001
*P*ΔN vs S	.742	1	.897	.103
*P*ΔP vs S	<.001	<.001	<.001	<.001

7-ICS = 7-indicator completeness score, GQS = Global Quality Score, JAMA = Journal of the American Medical Association, M = median, mDISCERN = modified DISCERN, Q1 = first quartile, Q3 = third quartile, SD = standard deviation.

* *P*-value of Kruskal–Wallis test; Δ*P*-value of Dunn’s post hoc test with Holm correction.

In the adjusted binary models, TikTok was associated with higher GQS (OR, 2.26; 95% CI: 1.27–4.02; *P* = .006) and lower 7-ICS (OR, 0.26; 95% CI: 0.13–0.50; *P* < .001), but was not associated with JAMA or mDISCERN (Table 4). Specialist-physician uploader status was associated with higher GQS (OR, 5.70; 95% CI: 3.17–10.26), JAMA (OR, 27.15; 95% CI: 10.79–68.33), mDISCERN (OR, 6.35; 95% CI: 2.95–13.66), and 7-ICS (OR, 10.10; 95% CI: 5.34–19.11; all *P* < .001). Longer video duration was also associated with higher scores for all 4 outcomes. Likes and comments were associated with JAMA, while shares were associated with 7-ICS, although the corresponding per-unit effect estimates were small.

**Table 4:** Multivariable ordinal logistic regression analysis using binary uploader classification.

Variables	GQS		JAMA		mDISCERN		7-ICS	
	*P*	OR (95% CI)	*P*	OR (95% CI)	*P*	OR (95% CI)	*P*	OR (95% CI)
Uploader								
Other uploaders (Ref.)		1.00		1.00		1.00		1.00
Specialist physicians	<.001	5.70 (3.17–10.26)	<.001	27.15 (10.79–68.33)	<.001	6.35 (2.95–13.66)	<.001	10.10 (5.34–19.11)
Platform								
Bilibili (Ref.)		1.00		1.00		1.00		1.00
TikTok	.006	2.26 (1.27–4.02)	.154	1.61 (0.84–3.08)	.713	1.14 (0.58–2.24)	<.001	0.26 (0.13–0.50)
Video length	<.001	1.01 (1.01–1.01)	.011	1.01 (1.01–1.01)	<.001	1.01 (1.01–1.01)	.001	1.01 (1.01–1.01)
Likes	.121	1.00 (1.00–1.00)	.033	1.01 (1.01–1.01)	.648	1.00 (1.00–1.00)	.114	1.00 (1.00–1.00)
Collections	.506	1.00 (1.00–1.00)	.579	1.00 (1.00–1.00)	.646	1.00 (1.00–1.00)	.604	1.00 (1.00–1.00)
Comments	.339	1.00 (1.00–1.00)	.002	0.99 (0.99–0.99)	.359	1.00 (1.00–1.00)	.254	1.00 (1.00–1.00)
Shares	.112	1.00 (1.00–1.00)	.575	1.00 (1.00–1.00)	.897	1.00 (1.00–1.00)	.044	1.01 (1.01–1.01)

7-ICS = 7-indicator completeness score, CI = confidence interval, GQS = Global Quality Score, JAMA = Journal of the American Medical Association, mDISCERN = modified DISCERN, OR = odds ratio.

In the 4-category models, TikTok remained associated only with higher GQS. Compared with patient uploaders, each nonpatient uploader category was associated with higher GQS and 7-ICS, while non-specialist and specialist physicians were also associated with higher mDISCERN. JAMA estimates for uploader categories could not be obtained because of quasi-complete separation (Table S5, Supplemental Digital Content 6).

### 3.5. Correlation analysis

Across all videos, duration correlated with 7-ICS (ρ = .41), GQS (ρ = .25), and mDISCERN (ρ = .20), but not JAMA. Engagement metrics were strongly intercorrelated (ρ = .74–.94) but generally unrelated to quality scores, while the 4 quality measures were moderately to strongly correlated (ρ = .34–.73; Fig. 2A, Supplemental Digital Content 7). On TikTok, duration correlated with GQS, mDISCERN, and 7-ICS, and collections correlated weakly with mDISCERN. On Bilibili, duration correlated only with 7-ICS; engagement metrics were not associated with quality measures (Fig. 2B, C, Supplemental Digital Content 7).

## 4. Discussion

Patients increasingly seek health information from online platforms, reshaping the traditional role of healthcare providers as the main source of medical guidance.^[29,30]^ Social media has become an important channel for health communication, with a large proportion of internet users searching for health-related information online.^[31,32]^ Anal fistula is a common anorectal disease that affects quality of life and is frequently discussed on short-video platforms. Such videos may provide convenient access to disease knowledge and partially meet public demand for health literacy.^[33]^ This study compared anal fistula-related videos on TikTok and Bilibili and evaluated differences in engagement, content coverage, quality, reliability, and uploader characteristics.

### 4.1. Quality of anal fistula short-video information

Overall, most videos did not reach high scientific standards, consistent with previous studies showing variable or suboptimal quality of health-related short videos.^[34]^ Similar findings have been reported for premature ovarian insufficiency^[35]^ and hepatocellular carcinoma^[36]^ videos on TikTok and Bilibili. The limited depth of many videos may be partly related to the short-video format, which constrains the breadth and detail of medical explanations.^[36]^

However, low-scoring videos are not necessarily without value. Some may still provide practical information that addresses patients’ immediate concerns. The main problem is the coexistence of useful information with incomplete or potentially misleading content, which may undermine credibility.^[27]^ Because video quality can influence users’ health-related decisions,^[11]^ greater participation by specialist physicians and stronger platform-level content review may help improve the accuracy and reliability of anal fistula-related information.

### 4.2. Imbalanced structure of short video content

The content distribution was imbalanced. Most videos discussed symptoms and treatment, whereas epidemiology and prevention were rarely covered. This may reflect both audience preferences and uploader strategies, as users are more likely to seek actionable information about symptoms and treatment than abstract preventive or epidemiological content. Nevertheless, the limited coverage of prevention suggests a missed opportunity for public health education. Collaboration between healthcare professionals and platforms could help create targeted content on modifiable risk factors, early recognition of perianal abscess, and timely care-seeking, thereby promoting earlier intervention and potentially reducing disease burden.^[37]^

### 4.3. Variations in short-video health content across platforms

TikTok videos were shorter and achieved higher engagement, whereas Bilibili videos had higher unadjusted 7-ICS scores and a higher JAMA score distribution. GQS and mDISCERN did not differ significantly. This is consistent with Wang et al, who reported that TikTok videos were shorter than Bilibili videos but generated higher user interaction rates.^[38]^ These differences may partly reflect variations in user demographics, video format, and platform mechanisms. TikTok’s younger user base and emphasis on brief, rapidly disseminated videos may facilitate interaction but limit the depth of disease-related information, whereas Bilibili allows longer videos that may better support comprehensive health education.^[39]^ This may help explain the higher unadjusted 7-ICS scores observed on Bilibili. Previous studies have also reported that longer videos tend to be associated with higher quality scores.^[40]^

After adjustment, the platform pattern remained nonuniform: TikTok was positively associated with GQS but inversely associated with 7-ICS in the binary uploader model and was not associated with JAMA or mDISCERN. Video duration, rather than platform alone, was consistently associated with all 4 outcomes. These findings should therefore be interpreted as cross-sectional associations rather than evidence that either platform inherently produces higher-quality content.

Most videos on both platforms lacked reference citations, reflecting a common weakness in short-video health communication. The absence of citations reduces traceability and academic rigor. Prior studies have similarly reported that many health-related videos on TikTok and Bilibili fail to reference scientific evidence.^[41]^ Potentially harmful or misleading information has also been reported in videos on atopic dermatitis^[42]^ and psoriasis.^[43]^ Platforms should therefore strengthen citation standards, expert review, and regulatory mechanisms to reduce misinformation and improve the reliability of health-related content.

### 4.4. Variations in short-video health content across uploaders

Specialist physicians were the predominant uploaders on both platforms. In the binary comparison, specialist-physician videos received more likes, collections, and shares, but not more comments, and had higher scores on all 4 evaluation measures. These findings are consistent with previous studies showing that videos created by specialist physicians or other healthcare professionals scored higher than those uploaded by private or nonmedical users.^[44,45]^ Professional expertise is important for health communication because expert-generated content is generally more scientific and standardized.^[46-50]^ Videos produced by specialist physicians may provide more accurate explanations, broader disease coverage, and clearer attribution, thereby serving as an important source of credible anal fistula-related information.

Patient-generated videos had the lowest quality scores and the highest median number of comments among the 4 uploader categories. These videos often focused on hospitalization costs, emotional experiences, and daily life rather than professional medical knowledge. Although their clinical content was limited, they may promote peer interaction and provide emotional support for patients with similar experiences.

Specialist-physician videos also had higher JAMA scores than institutional and patient-generated videos. This pattern may reflect more frequent reporting of author identity, hospital affiliation, department, or ownership information. However, disclosure practices should be standardized. Liu et al reported that certified creators receive greater platform support and recommended that specialist-physician uploaders apply for verification.^[51]^ Platforms, medical societies, and professional associations could further collaborate to establish evidence-based content standards and more transparent professional certification systems, thereby helping users identify reliable medical information.

### 4.5. The quality–popularity paradox in health videos

Correlation analysis showed strong positive associations among likes, comments, collections, and shares, consistent with Li et al.^[52]^ However, engagement metrics were generally not associated with quality scores, except for a weak correlation between collections and mDISCERN on TikTok. This finding is consistent with previous studies suggesting that user engagement is driven more by popularity than by content quality.^[18,53-55]^ Therefore, platforms should optimize content review and recommendation mechanisms to promote the dissemination of high-quality health education videos rather than relying primarily on engagement-based popularity signals.

## 5. Limitations

This study has several limitations. First, as a cross-sectional study, we acknowledge that short-video content is dynamic; therefore, our data represent a snapshot in time and may not fully reflect the most rapidly evolving trends. Second, our analysis was restricted to Chinese-language videos, which limits the generalizability of the findings to other regions. Cultural and linguistic differences, such as divergent clinical approaches to anal fistula management between Eastern and Western contexts, likely influence content creation. Future cross-cultural comparisons (e.g., between the international and Chinese versions of TikTok) could help reveal broader patterns in health information dissemination. Third, although we implemented a rigorous calibration process and achieved good inter-rater reliability, the use of subjective scoring scales inevitably introduces some degree of observer bias. Fourth, while selecting the top 200 videos is a method supported by previous literature and offers a reasonable balance between feasibility and relevance, this pragmatic threshold may not perfectly align with the actual scrolling behavior of every user. This strategy captures high-visibility content but may exclude less prominent yet potentially relevant videos. Finally, our reliance on the platforms’ default comprehensive rank means the sample is inherently influenced by proprietary algorithms. Since the specific weighting of factors, such as engagement, recency, and authority, remains undisclosed, our dataset reflects content prioritized by the platforms as salient rather than a statistically representative sample of the entire repository of anal fistula-related videos.

## 6. Conclusion

This cross-sectional study evaluated 333 anal fistula-related videos on TikTok and Bilibili using GQS, mDISCERN, JAMA, and 7-ICS. Overall video quality was suboptimal. Bilibili showed higher unadjusted JAMA and 7-ICS results, whereas adjusted platform associations were nonuniform: TikTok was positively associated with GQS but inversely associated with 7-ICS in the binary uploader model and was not significantly associated with JAMA or mDISCERN. Specialist-physician uploader status and longer video duration were independently associated with higher scores across all 4 outcomes. Patients should interpret short-video information cautiously and supplement it with professional medical consultation.

## Author contributions

**Conceptualization:** Xizhe Du.

**Data curation:** Xizhe Du, Shuting Pan, Xia Xiao, Yingru Zhuang.

**Methodology:** Xizhe Du.

**Project administration:** Yingru Zhuang.

**Writing – original draft:** Xizhe Du, Shaopeng Huang.

**Writing – review & editing:** Qinghua Ye, Jiayi Xia, Yingru Zhuang.














