# Supplementary material for: Quality and content evaluation of anal fistula information on TikTok and Bilibili: A cross-sectional study
Source: Medicine (Baltimore). 2026 Aug 14;105(33):e50130. doi: 10.1097/MD.0000000000050130 (PMC13480743; doi:10.1097/MD.0000000000050130)
Supplement: Supplementary file 2 [file medi-105-e50130-s002.docx]

Supplementary Table 2. Modified DISCERN (mDISCERN) quality criteria.

**Reliability Score**

1. Is the video clear, concise, and understandable?

2. Are valid sources cited?

3. Is the content presented balanced and unbiased?

4. Are additional sources of content listed for patient reference?

5. Are areas of uncertainty mentioned?

*Each item was assigned 1 point for “yes” and 0 points for “no.”
