# Supplementary material for: Quality and content evaluation of anal fistula information on TikTok and Bilibili: A cross-sectional study
Source: Medicine (Baltimore). 2026 Aug 14;105(33):e50130. doi: 10.1097/MD.0000000000050130 (PMC13480743; doi:10.1097/MD.0000000000050130)
Supplement: Supplementary file 3 [file medi-105-e50130-s003.docx]

Supplementary Table 3. Journal of the American Medical Association (JAMA) benchmark criteria.

| Score* | Description | |
| --- | --- | --- |
| 1 score | Authorship | Author and contributor credentials and their affiliations should be provided. |
| 1 score | Attribution | Clearly lists all copyright information and states references and sources for content. |
| 1 score | Currency | Initial date of posted content and subsequent updates to content should be provided. |
| 1 score | Disclosure | Conflicts of interest, funding, sponsorship, advertising, support, and video ownership  should be fully disclosed. |

*Each criterion was scored separately, with 1 point assigned when the criterion was met; the total score ranged from 0 to 4.
