# Supplementary material for: Quality and content evaluation of anal fistula information on TikTok and Bilibili: A cross-sectional study
Source: Medicine (Baltimore). 2026 Aug 14;105(33):e50130. doi: 10.1097/MD.0000000000050130 (PMC13480743; doi:10.1097/MD.0000000000050130)
Supplement: Supplementary file 4 [file medi-105-e50130-s004.docx]

Supplementary Table 4. Video length and engagement metrics across four uploader categories.

|  | **Video length, M (Q₁, Q₃)** | **Likes, M (Q₁, Q₃)** | **Collections, M (Q₁, Q₃)** | **Comments, M (Q₁, Q₃)** |  | **Shares, M (Q₁, Q₃)** |
| --- | --- | --- | --- | --- | --- | --- |
| Institution(n=40) | 72.00 (41.75,150.25) | 90.50 (13.25,695.25) | 50.00 (8.50,271.00) | 20.00 (1.00,208.00) | | 57.00 (8.75,265.50) |
| Non-specialist physician(n=23) | 85.00 (55.50,355.50) | 72.00 (21.00,439.00) | 77.00 (8.00,361.50) | 8.00 (0.50,39.50) | | 16.00 (5.50,141.50) |
| Patient(n=97) | 78.00 (10.00,175.00) | 207.00 (53.00,1176.00) | 28.00 (9.00,204.00) | 341.00 (107.00,705.00) | | 36.00 (8.00,287.00) |
| Specialist(n=173) | 54.00 (37.00,89.00) | 617.00 (56.00,1973.00) | 121.00 (13.00,477.00) | 101.00 (10.00,421.00) | | 148.00 (8.00,1219.00) |
| *P** | .008 | .011 | .039 | <.001 | | .029 |
| *P*ΔI vs N | .486 | .845 | 1 | .504 | | 1 |
| *P*ΔI vs P | .217 | .402 | 1 | <.001 | | 1 |
| *P*ΔI vs S | .217 | .044 | 1 | .014 | | .480 |
| *P*ΔN vs P | .025 | .402 | 1 | <.001 | | 1 |
| *P*ΔN vs S | .023 | .093 | 1 | .011 | | .194 |
| *P*ΔP vs S | .947 | .402 | .025 | <.001 | | .096 |

M = median; Q1 = first quartile; Q3 = third quartile; I = institution; N = non-specialist physician; P = patient; S = specialist physician.

*P-value of Kruskal–Wallis test; ΔP-value of Dunn post-hoc test with Holm correction
