# Supplementary material for: Quality and content evaluation of anal fistula information on TikTok and Bilibili: A cross-sectional study
Source: Medicine (Baltimore). 2026 Aug 14;105(33):e50130. doi: 10.1097/MD.0000000000050130 (PMC13480743; doi:10.1097/MD.0000000000050130)
Supplement: Supplementary file 5 [file medi-105-e50130-s006.docx]

Supplementary Table 5. Multivariable ordinal logistic regression analysis using four-category uploader classification.

|  | **GQS** | | **JAMA** | | **mDISCERN** | | **7- ICS** | |
| --- | --- | --- | --- | --- | --- | --- | --- | --- |
| **Variables** | ***P*** | **OR (95% *CI*)** | ***P*** | **OR (95% *CI*)** | ***P*** | **OR (95% *CI*)** | ***P*** | **OR (95% *CI*)** |
| **Uploader** |  |  |  |  |  |  |  |  |
| Patients (Ref.) |  | 1.00 | - | - |  | 1.00 |  | 1.00 |
| Institutions | <.001 | 22.68 (6.28 - 81.93) | † | † | .212 | 3.11 (0.52 - 18.41) | .003 | 4.72 (1.67 - 13.35) |
| Non-specialist physicians | <.001 | 23.07 (5.76 - 92.30) | † | † | .001 | 13.96 (2.79 - 69.94) | .009 | 6.42 (1.59 - 25.85) |
| Specialists | <.001 | 29.57 (10.15 - 86.12) | † | † | <.001 | 16.72 (4.76 - 58.77) | <.001 | 16.19 (8.12 - 32.27) |
| **Platform** |  |  |  |  |  |  |  |  |
| Bilibili (Ref.) |  | 1.00 |  | 1.00 |  | 1.00 |  | 1.00 |
| TikTok | <.001 | 3.91 (2.05 - 7.44) | .102 | 1.76 (0.89 - 3.49) | .404 | 1.35 (0.67 - 2.74) | .055 | 0.48 (0.23 - 1.02) |
| **Video length** | <.001 | 1.01 (1.01 - 1.01) | .060 | 1.00 (1.00 - 1.00) | .004 | 1.01 (1.01 - 1.01) | <.001 | 1.01 (1.01 - 1.01) |
| **Likes** | .122 | 1.00 (1.00 - 1.00) | .101 | 1.00 (1.00 - 1.00) | .479 | 1.00 (1.00 - 1.00) | .183 | 1.00 (1.00 - 1.00) |
| **Collections** | .497 | 1.00 (1.00 - 1.00) | .652 | 1.00 (1.00 - 1.00) | .417 | 1.00 (1.00 - 1.00) | .592 | 1.00 (1.00 - 1.00) |
| **Comments** | .623 | 1.00 (1.00 - 1.00) | .005 | 0.99 (0.99 - 0.99) | .138 | 1.00 (1.00 - 1.00) | .372 | 1.00 (1.00 - 1.00) |
| **Shares** | .304 | 1.00 (1.00 - 1.00) | .833 | 1.00 (1.00 - 1.00) | .459 | 1.00 (1.00 - 1.00) | .117 | 1.00 (1.00 - 1.00) |

*CI* = confidence interval; OR = odds ratio; 7-ICS = 7-indicator completeness score; GQS = Global Quality Score; JAMA = Journal of the American Medical Association; mDISCERN = modified DISCERN.

† **=** Estimates for the 'Uploader' variable in the JAMA model were not available due to quasi-complete separation of data (zero cell counts in specific subgroups), which prevented model convergence for this specific variable
